# Molecular Engineering of the Helminth TGF‐β Mimetics, TGM1 and TGM4, Reveals a Novel Antagonist of TGF‐β Signaling in Fibroblasts

**DOI:** 10.1096/fj.202503194R

**Published:** 2026-03-31

**Authors:** Kyle T. Cunningham, Claire Ciancia, Tiffany Campion, Maarten van Dinther, Nadia Davis, Anja Duffy, Anna L. L. Heawood, Luke Power, Anna Sanders, Shashi P. Singh, Danielle J. Smyth, Elizabeth Thompson, Ruby White, Andrew P. Hinck, Peter ten Dijke, Rick M. Maizels

**Affiliations:** ^1^ Centre for Parasitology, School of Infection and Immunity University of Glasgow Glasgow UK; ^2^ Champalimaud Centre for the Unknown Lisbon Portugal; ^3^ Erasmus MC Department of Viroscience Rotterdam the Netherlands; ^4^ INSERM University of Toulouse Toulouse France; ^5^ Oncode Institute and Department of Cell and Chemical Biology University of Leiden Leiden the Netherlands; ^6^ Division of Immunology, Immunity to Infection and Respiratory Medicine University of Manchester Manchester UK; ^7^ Department of Neuroscience NYU Langone Health Grossman School of Medicine New York New York USA; ^8^ Department of Biological Sciences Birla Institute of Technology and Science Pilani Rajasthan India; ^9^ Division of Cell Signalling and Immunology University of Dundee Dundee UK; ^10^ Department of Structural Biology University of Pittsburgh Pittsburgh Pennsylvania USA

**Keywords:** CD44, chimeric proteins, fibroblast, *Heligmosomoides polygyrus*, signaling, T cell, TGF‐β, TGF‐β mimic, TGF‐β receptor

## Abstract

The transforming growth factor‐β (TGF‐β) mimics TGM1 and TGM4 are modular proteins expressed by the parasite *Heligmosomoides polygyrus*. Each comprises 5 complement control protein (CCP) domains binding the TGF‐β receptors TGFBR1 (Domains 1 and 2) and TGFBR2 (Domain 3), whereas Domains 4 and 5 bind cell surface co‐receptors including CD44. Despite sharing 86% amino acid identity, TGM1 and TGM4 differ functionally in key respects of affinity for mammalian TGFBRs and interactions with cell surface co‐receptors. Although both induce T cell expression of the transcription factor Foxp3, TGM1 activates intracellular SMAD signaling in fibroblasts, whereas conversely, TGM4 blocks SMAD responses to TGF‐β or TGM1 in fibroblasts. By recombining and/or deleting domains, we identified that the TGFBR1‐binding Domains 1 and 2 of TGM4 confer antagonist activity, which is dependent on the CD44 co‐receptor interacting domains (Domains 4 and 5); antagonism is enhanced but not fully dependent on binding to TGFBR2. TGMs lack interchain disulfide bonds, in distinction to TGF‐β, which is a covalently linked dimeric cytokine. Dimerization of TGM4 as an Fc fusion protein converted it from an inhibitor to an activator of SMAD signaling in fibroblasts and enhanced Foxp3 induction, indicating that high avidity interactions (either monomer/dimer on T cells or only dimer on fibroblasts) drive TGF‐β signaling. However, when TGM4 is below an avidity threshold, it binds receptors in an antagonistic mode. These results advance our understanding of the mechanism of action of these parasite‐encoded products and uncover new tools modulating TGF‐β functions in health and disease.

## Graphical abstract

The helminth TGF‐β mimic TGM4 activates SMAD signaling in macrophages and T cells through canonical TGF‐β receptors and the CD44 co‐receptor, through Domains 1–5 (D1‐5). In fibroblasts, which express lower levels of CD44, TGM4 binds but does not transduce a signal; furthermore, it inhibits activation by agonist ligands such as TGF‐β itself, in a manner that depends on the TGFBR1‐binding Domains 1 and 2 (D1, D2). However, when dimerized through a fusion with Fc, TGM4 is able to activate signaling in all 3 types of cell.

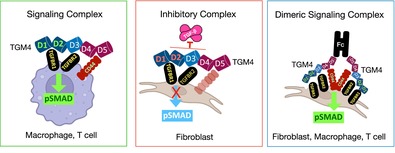

## 1 Introduction

Transforming growth factor‐β (TGF‐β) is a multipotent cytokine that regulates differentiation, activation, and regulation of the immune system [1, 2]. TGF‐β elicits its pleiotropic effects by specifically interacting with two related single transmembrane serine/threonine kinase receptors, that is, TGFBR1 and TGFBR2, in a heteromeric complex [3]. Although both TGFBRs are essential for signaling, TGFBR1 acts downstream of TGFBR2 [4]. Co‐receptors, such as betaglycan, are more indirectly involved, for example, by increasing the cellular avidity that combines the affinities of individual receptor‐ligand interactions [5]. Intracellular TGF‐β receptor signaling is mediated by SMAD transcription factors; TGFBR2‐mediated activation of TGFBR1 stimulates the phosphorylation of SMAD2 and SMAD3, which form heteromeric complexes with SMAD4. These heteromeric SMAD complexes can accumulate in the nucleus, where they act as transcription factors to regulate gene responses [6, 7].

Some infectious agents have exploited the broadly immune‐suppressive properties of TGF‐β to further their tenure in the host [8]. Thus, the helminth parasite *Heligmosomoides polygyrus* expresses a family of TGF‐β mimics (TGMs) with novel protein structures unrelated to TGF‐β itself that activate the mammalian TGF‐β receptor signaling pathway [9, 10, 11, 12].

The TGMs are modular proteins with multiple domains related to the complement control protein (CCP) or Sushi family, which have evolved through domain duplication and diversification within the *H. polygyrus* lineage [9, 13, 14]. TGM1 and TGM4 are both 5‐domain proteins, in which Domains 1 and 2 (D1‐2) bind TGF‐β receptor I (TGFBR1) and Domain 3 (D3) binds TGFBR2, albeit with differing affinities [10, 15]. In addition, Domains 4 and 5 (D4‐5) bind cell surface co‐receptors such as CD44 [16], raising the avidity of ligands for the signaling complex. Optimal cellular responsiveness requires expression of co‐receptors, which are differentially expressed on different cell types, conferring cell specificity to signaling by TGM proteins. For example, TGM1 but not TGM4 can activate fibroblasts, whereas TGM4 is most efficacious on myeloid cells [15].

A further family member, TGM6, lacks D1‐2 and is unable to drive TGF‐β signaling in any cell type tested [12]; moreover, TGM6 can competitively inhibit signaling by TGM1 or TGF‐β in a manner dependent on the co‐receptor specific to its D4‐5. Hence, depending on the configuration of domains, TGMs may act as stimulatory or inhibitory agents, and do so by combining interactions with both conventional TGF‐β receptors and other cell surface components.

A further notable difference between TGMs and TGF‐β is that the former appears to be monomeric, whereas the latter is covalently dimerized through a disulfide bond, such that although TGMs engage a single TGFBR1/TGFBR2 heterodimeric receptor, TGF‐β homodimers assemble a heterotetrameric complex of two TGFBR1 and two TGFBR2 receptor subunits as two independently binding TGFBR1‐TGFBR2 heterodimers [17, 18, 19, 20]. Although these reports address the question of whether ligation by TGF‐β of one TGFBR1‐TGFBR2 heterodimeric pair differs in efficacy compared to ligation of two TGFBR1‐TGFBR2 heterodimeric pairs [17], the effects of dimerization by molecular engineering of the parasite ligand have not yet been tested.

In this study, we investigated and modified the TGM1 and TGM4 proteins to determine which domains best modulate mammalian TGFBR/SMAD signaling, if conversion to divalent ligands boosts potency, and whether these ligands can be converted into potent TGFBR/SMAD signaling inhibitors by deleting certain components. In this fashion, we have advanced both our understanding of these parasite‐encoded products and the search for new tools for the long‐sought goal of modulating TGF‐β functions in health and disease [21].

## 2 Materials and Methods

### 2.1 Expression of TGM1 and TGM4 Recombinant Proteins

The sequences for *H. polygyrus bakeri* TGM1 and TGM4 are available from NCBI (Accession Numbers MG099712 and MG429739), and comprise 5 distinct domains as presented in Table S1. Recombinant proteins and chimeric constructs were produced from synthetic genes prepared commercially by Twist Bioscience or GeneArt (Thermo Fisher), supplied in pTwist‐Amp or pMK plasmids, respectively, with flanking *Asc*I and *Not*I restriction sites. For proteins without Fc fusion partners, inserts encoding the domains indicated in Table S1 were removed by digestion with *Asc*I and *Not*I and subcloned into the mammalian expression vector pSecTag2A using the same restriction sites, resulting in an additional 7 *N*‐terminal amino acids (aa) (DAANPPG) after cleavage of the vector‐encoded signal peptide, and an additional 28 vector‐encoded *C*‐terminal amino acids including a final 6‐Histidine tail. Following sequence verification, purified plasmids were used to transfect HEK293T or Expi293F cells as previously described [13]. Proteins were purified from the conditioned media on nickel‐loaded HisTrap Excel chelating columns (Cytiva 17371205) and eluted with a 0.0–0.5 M imidazole gradient. All proteins and constructs used in this study are summarized in Table S2.

### 2.2 Construction of Fc‐Dimerized TGM Proteins

Dimers were constructed using the pFUSE‐hIgG1‐Fc2 plasmid (InvivoGen), with or without an additional 45‐bp linker encoding 3 copies of the flexible GGGGS peptide linker. Inserts were cloned into the *Nco*I and *Nhe*I sites. TGM‐Fc fusion proteins were purified on a 1 mL HiTrap Protein G HP column (Cytiva 17040403).

### 2.3 Cell Line Cultures

Human HEK293T (CRL‐1573) cells were purchased from the American Type Culture Collection (ATCC), cultured in Dulbecco's modified Eagle medium (DMEM; Thermo Fisher Scientific; 41965062) supplemented with 10% fetal bovine serum (FBS; Thermo Fisher Scientific; 16000044) and 100 U/mL penicillin, 100 ug/mL streptomycin (Thermo Fisher Scientific; 15140163). Expi293F cells were purchased from Gibco/Thermo Fisher, transfected with plasmid DNA with ExpiFectamine, and cultured for 4–5 days in OptiMEM Medium (Gibco/Thermo Fisher). MFB‐F11 embryonic fibroblasts from *Tgfb1* ^*−/−*^ mice, stably transfected with a reporter expressing alkaline phosphatase under a SMAD3‐responsive promoter [22] were maintained in DMEM with 10% FBS. Generation of NIH‐3T3 fibroblasts (CRL‐1658) containing the fluorescent‐based TGF‐β/SMAD3 transcriptional reporter, CAGA‐MLP‐dynGFP, was previously described [12]. All cell lines were maintained in a 5% CO_2_, 37°C humidified incubator, tested monthly for mycoplasma contamination, and checked for authenticity by short tandem repeat (STR) profiling.

### 2.4 Western Blot Assays

Western blot assays for SMAD phosphorylation were performed as previously described [15, 23]. Briefly, following ligand treatment, cell lysates were analyzed on 4%–12% bis‐tris SDS‐PAGE gels and transferred onto a nitrocellulose membrane using iBlot2 (Invitrogen, IB21001). Membranes were treated in 5% non‐fat milk blocking solution for 1 h and incubated with primary rabbit polyclonal anti‐SMAD2/3 (Cell Signaling Technology #3102, #5678), or with rabbit monoclonal antibody D27F4 to phospho‐SMAD2 (Ser465/467)/SMAD3 (Ser423/425) rabbit mAb (Cell Signaling Technology, Cat. No. 8828), each at 1:1000 in 5% BSA containing TBST, overnight at 4°C. Following three 5‐min washes with TBST, fluorescent‐conjugated secondary polyclonal goat anti‐rabbit IgG (DyLight 680, Invitrogen SA535571) diluted 1:10 000 in 5% BSA containing TBST was used to detect binding by the Odyssey CLx Imaging System (LI‐COR Biosciences).

### 2.5 TGF‐β Reporter BioAssays

TGF‐β receptor/SMAD3 signaling was measured with an MFB‐F11 cell assay [22] as previously described [9, 13]. Briefly, cells from confluent cultures were detached with trypsin and resuspended at 8 × 10^5^ cells/mL in DMEM, 2.5% FBS, 100 U/mL penicillin, 100 μg/mL streptomycin, and 2 mM L‐glutamine; 50 μL (4 × 10^4^ cells) was added to each well of a 96‐well flat‐bottomed plate. Test proteins were then added in a 50 μL volume and incubated for 24 h at 37°C. Subsequently, 20 μL of supernatant was aspirated from each well, added to an enzyme‐linked immunosorbent assay (ELISA) plate (Nalge Nunc International, USA) with 180 μL of reconstituted Sigma FastTM p‐nitrophenyl phosphate substrate, and incubated at room temperature in the dark for up to 18 h. Plates were read at 405 nm on an Emax precision microplate reader (Molecular Devices, USA).

In addition, TGF‐β receptor/SMAD3 signaling in NIH‐3T3 fibroblasts was measured using the CAGA‐adenovirus major late promoter (MLP)‐dynamic (dyn) green fluorescent protein (GFP) transcriptional reporter, as previously described [23]. Briefly, NIH‐3T3 cells containing the CAGA‐dynGFP reporter were seeded in 96‐well plates. The next day, cells were treated sequentially with ligands as indicated in full culture media, and were placed in the IncuCyte S3 live‐cell imaging analysis system (Sartorius). The cells were subsequently imaged every 3 h for a period of 48 h. Fluorescence intensity was analyzed using the IncuCyte software.

### 2.6 Foxp3^+^ Treg Induction Assay

Single cell suspensions of naïve BALB/c or Foxp3‐green fluorescent protein (GFP) BALB/c transgenic [24] mouse splenocytes were prepared and incubated for 2 min in red blood cell lysis buffer (Sigma) before washing and resuspending in RPMI1640 containing HEPES (Gibco) 2 mM L‐glutamine, 100 U/mL penicillin, 100 μg/mL streptomycin (Gibco), 10% heat‐inactivated FBS (Gibco), and 50 nM 2‐mercaptoethanol (Gibco). Naïve CD4^+^ T cells were then isolated by magnetic sorting using the mouse naïve CD4^+^ T cell isolation kit on the AutoMACS system (Miltenyi, Germany) as per the manufacturer's instructions. For Foxp3 induction, flat‐bottomed 96‐well plates (Corning, USA) pre‐coated with 10 μg/mL of anti‐CD3 (eBioscience), were populated with 2 × 10^5^ cells per well in media supplemented with 400 U/mL interleukin (IL)‐2 (Miltenyi). Cells were incubated at 37°C, 5% CO_2_ for at least 72 h before flow cytometric analysis. For inhibition of TGFBR2, small molecule ITD1 was used, for TGFBRI (activin receptor‐like kinase (ALK5)), 5 μM SB431542 [25, 26] (Tocris Bioscience, UK), SB525334 [27] (Tocris Bioscience, UK), and for SMAD3 SIS3 [28] was added, with dimethylsulfoxide (DMSO) added to vehicle control wells.

### 2.7 Flow Cytometry and Phosflow Analyses

For the determination of CD44 expression, small intestinal tissues from female C57BL/6 mice were harvested and opened longitudinally before being washed in PBS and cut into pieces. Tissues were stored at 4°C in calcium/magnesium‐free HBSS (ThermoFisher) supplemented with 10% FBS. For lamina propria leukocyte (LPL) isolation, tissues were transferred to HBSS supplemented with 2 mM EDTA (ThermoFisher) and incubated at 37°C in a shaking incubator at 220 rpm for 15 min before repeating this step twice in fresh EDTA‐HBSS for a total of 3 EDTA‐HBSS washes. SI tissue was then digested at 37°C for 15 min in a shaking incubator at 220 rpm with 0.5 mg/mL collagenase VIII (Sigma‐Aldrich) in complete R10 medium (RPMI‐1640 supplemented with 10% FBS, 100 U/mL penicillin, 100 μg/mL streptomycin, 2 mM L‐glutamine, and 50 μM β‐mercaptoethanol—all Gibco). Digested samples were passed through 100 μm and 40 μm filters to obtain single cell suspensions. Cells were then centrifuged twice in R10 at 400 *g* for 10 min at 4°C before staining for flow cytometry, using anti‐CD44 antibody (Clone IM7, BioLegend). CD4^+^ T cells were identified as CD45^+^, CD3^+^CD4^+^CD19^−^; macrophages as CD45^+^CD11b^+^CD64^+^Ly6C^+^MHC‐II^+^; and fibroblasts as CD45^−^CD31^−^ESAM^−^Podoplanin^+^, staining 2 × 10^6^ cells per sample with surface antibodies for flow cytometry analysis. All staining steps were carried out in PBS supplemented with 2% FBS and 2 mM EDTA (FACS buffer). Cells were first incubated with Fixable Viability Dye (Invitrogen) and TruStain FcX (anti‐mouse CD16/32) antibody (BioLegend) for 20 min at 4°C to exclude dead cells and minimize non‐specific antibody binding, respectively. Cells were washed at 400 *g* for 5 min at 4°C and then stained with a combination of surface antibodies for 30 min at 4°C. Cells were washed again and fixed in Fixation Buffer (BioLegend) for 30 min at 4°C and then washed and stored in FACS buffer at 4°C until acquisition.

For analysis of Foxp3 expression and SMAD phosphorylation by flow cytometry, cells were harvested from BALB/c or Foxp3‐GFP BALB/c transgenic mice. Cells were washed with phosphate‐buffered saline (PBS) twice and stained with Fixable Viability Dye eFluor 506 (eBioscience) in the dark for 15 min at room temperature. Cells were then washed twice with PBS and resuspended with TruStain FcX PLUS (anti‐mouse CD16/32) antibody to block unspecific binding. Cells were left to block in the dark for 15 min at room temperature. Following washing with PBS, cells were stained with PerCP/Cy5.5‐conjugated antibodies to CD4 (Biolegend) for 30 min on ice, in the dark in flow buffer (PBS, 0.5% BSA, 5 mM EDTA). After incubation, cells were washed twice with flow buffer. Cells were resuspended and assessed using a BD FACSCelesta Cell Analyzer (BD), and data were analyzed by FlowJo analysis software. For Phosflow assays, before staining, cells were first fixed with pre‐warmed Fix/Lyse (BD) to prevent further changes to sensitive signaling pathways. Cells were left to incubate at 37°C for 12 min before centrifugation. The fixed cells were then permeabilized with chilled Perm Buffer III for 30 min on ice. Cells were then washed with flow buffer and stained for 1 h at room temperature for CD4 (BioLegend), pSMAD1/5, and pSMAD2/3 (BD). Finally, cells were washed thoroughly and resuspended in a flow buffer for analysis on the cytometer.

### 2.8 Statistical Analyses

GraphPad Prism was used for statistical analyses detailed in each figure legend.

## 3 Results

### 3.1 Inhibitory Activity of TGM4 on Activation of Fibroblasts by TGF‐β and TGM1

**FIGURE 1:**
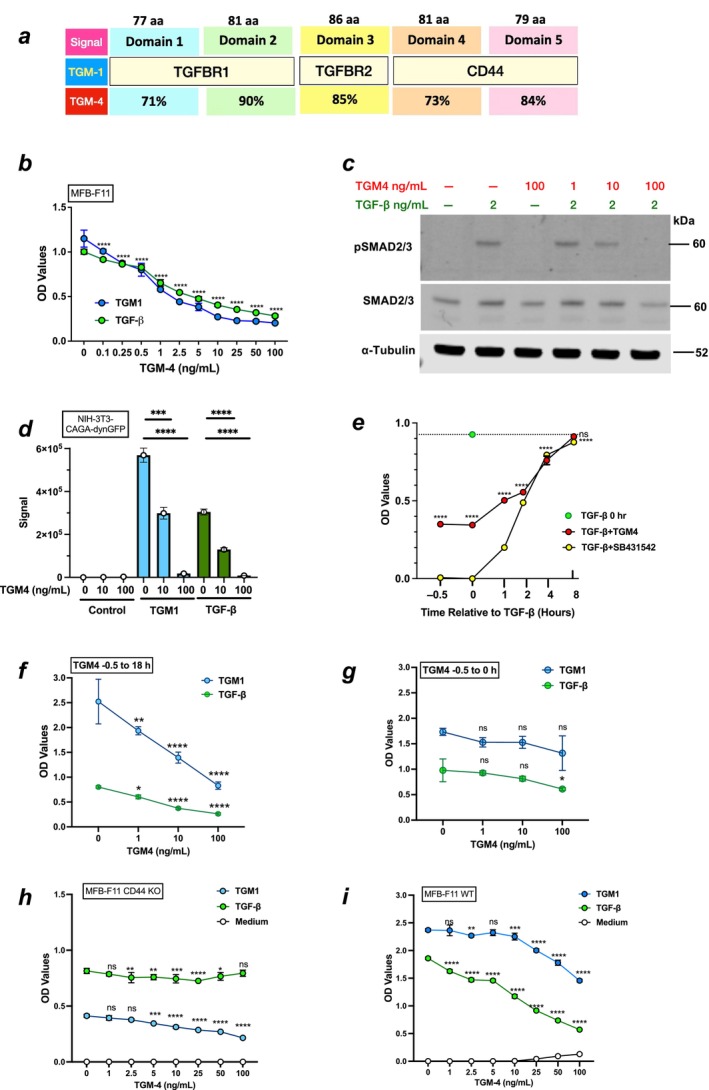
Inhibition of TGM1 and TGF‐β Signaling by TGM4. (a) Schematic representation of the 5 domains of TGM1 and TGM4, indicating amino acid lengths, receptor specificity, and percentage amino acid identity between the two proteins in each domain. (b) Inhibition of TGF‐β (5 ng/mL) and TGM1 (10 ng/mL) signaling in MFB‐F11 transcriptional reporter murine fibroblasts by increasing concentrations of TGM4, as measured by the release of alkaline phosphatase. Data are means ± SD, *n* = 3 from one of two replicate experiments, analyzed by two‐way ANOVA with Dunnett's multiple comparison test; showing statistics comparing TGF‐β responses with TGM4 versus no TGM4. ****p < 0.0001. (c) Inhibition of SMAD2/3 phosphorylation in MFB‐F11 fibroblasts as measured by Western blotting of cell lysates following stimulation with TGF‐β and/or TGM4 (2.5 ng/mL) as indicated. Data are from one of three independent experiments. α‐Tubulin, loading control. (d) Inhibition of signaling in murine NIH 3 T3 fibroblasts expressing dynGFP under a CAGA promoter responsive to SMAD3 signaling, following stimulation with TGF‐β (1 ng/mL) or TGM1 (2.5 ng/mL) in the presence of the indicated doses of TGM4. Data are means ± SD, *n* = 3 from 1 of three replicate experiments, analyzed by unpaired *t* test. ***p < 0.001, ****p < 0.0001. (e) Kinetics of inhibition of 5 ng/mL TGF‐β signaling in MFB‐F11 transcriptional reporter fibroblasts exposed to 100 ng/mL TGM4 or the pharmacological TGFBR1‐like kinase inhibitor SB431542 (5 μM) at different time points relative to administration of TGF‐β. Data are mean ± SD, *n* = 3 from one of three replicate experiments analyzed by two‐way ANOVA with Sidak's multiple comparison test; showing statistics comparing TGF‐β responses with TGM4 versus no TGM4. ****p < 0.0001; ns, not significant (*p* > 0.05). (f and g) Inhibition of signaling in MFB‐F11 transcriptional reporter fibroblasts receiving the indicated doses of TGM4 at 30 min prior to 5 ng/mL TGF‐β or 10 ng/mL TGM1, without (f) and with (g) washing and removal of TGM4 at the indicated doses. Data are mean ± SD, *n* = 3 from one of three replicate experiments, analyzed by two‐way ANOVA with Dunnett's multiple comparison test; showing statistics comparing TGM1 or TGF‐β responses with TGM4 versus no TGM4. *p < 0.05, **p < 0.01, ****p < 0.0001; ns, not significant (*p* > 0.05). (h and i) TGM4 treatment of CD44 KO MFB‐F11 transcriptional reporter cells (h) does not inhibit TGF‐β signaling, whereas it is highly inhibitory in WT cells (i); cells were stimulated with 5 ng/mL TGF‐β or 10 ng/mL TGM1. Data are mean ± SD, *n* = 3 from one of three replicate experiments, analyzed by two‐way ANOVA with Dunnett's multiple comparison test; showing statistics comparing TGM1 or TGF‐β responses with TGM4 versus no TGM4. *p < 0.05, **p < 0.01, ***p < 0.001, ****p < 0.0001; ns, not significant (*p* > 0.05).

TGM1 and TGM4 each comprise 5 homologous ~80‐aa domains sharing 71%–90% amino acid identity (Figures 1a and S1, Table S1); sequence differences account for significant variation in the affinity for TGM1 and TGM4 host (co‐)receptors, TGFBR1, TGFBR2, and CD44 (Figure S1b). As a result of these structural differences, TGM4 fails to induce TGFBR/SMAD signaling in fibroblasts despite demonstrable affinity for the canonical TGFBRs and ability to signal in T cells and macrophages; TGM1, however, can fully activate signaling in fibroblasts [15].

As TGM4 can bind both TGFBRs but fails to activate fibroblasts, we first asked whether it was able to antagonize TGM1‐ or TGF‐β‐induced SMAD signaling in these cells. Mouse MFB‐F11 SMAD3 transcriptional reporter fibroblasts [22] were incubated with TGM4 in the presence or absence of a stimulatory concentration of TGM1. As presented in Figure 1b, TGM4 inhibited the TGM1‐induced SMAD3 response in a dose‐dependent manner. In addition, TGM4 constrained the response to mammalian TGF‐β to a similar degree at all tested doses (Figure 1b). To confirm these observations, we also probed the cell lysates of TGF‐β‐stimulated cells in the absence or presence of TGM4 with antibodies to p‐SMAD2/3 and again found that higher concentrations of TGM4 ablated the response to TGF‐β (Figure 1c). Similar results were obtained with an alternative reporter cell line of mouse NIH 3T3 fibroblasts expressing GFP under a SMAD3/4‐responsive CAGA element, in which responses to either TGM1 or TGF‐β were completely suppressed by 100 ng/mL TGM4 (Figure 1d).

In these experiments, cells were treated with TGM4 for 30 min before the addition of TGM1 or TGF‐β. Inhibition was equally effective if the agonist and antagonist were administered at the same time and became progressively less marked if TGM4 was added up to 4 h later than TGF‐β (Figure 1e). We similarly tested the pharmacological inhibitor, SB431542, which selectively targets TGFBR1‐like kinases [25]. SB431542 showed more complete suppression than TGM4 when co‐administered, and a similar loss of efficacy as addition to cells was delayed (Figure 1e). TGM4 inhibition, moreover, required continuous presence with cells, as pre‐treatment for 30 min and washing before agonist addition showed barely any reduction in TGF‐β/SMAD signaling (Figure 1f,g).

TGM4, like TGM1, binds to the co‐receptor CD44 through D4 and −5 [15, 16]. We genetically deleted CD44 from MFB‐F11 cells and found that TGM4 was no longer able to inhibit TGF‐β/SMAD signaling, albeit responses in these modified cells are relatively muted, in particular to TGM1 (Figure 1h,i).

### 3.2 Mutation or Deletion of D3

**FIGURE 2:**
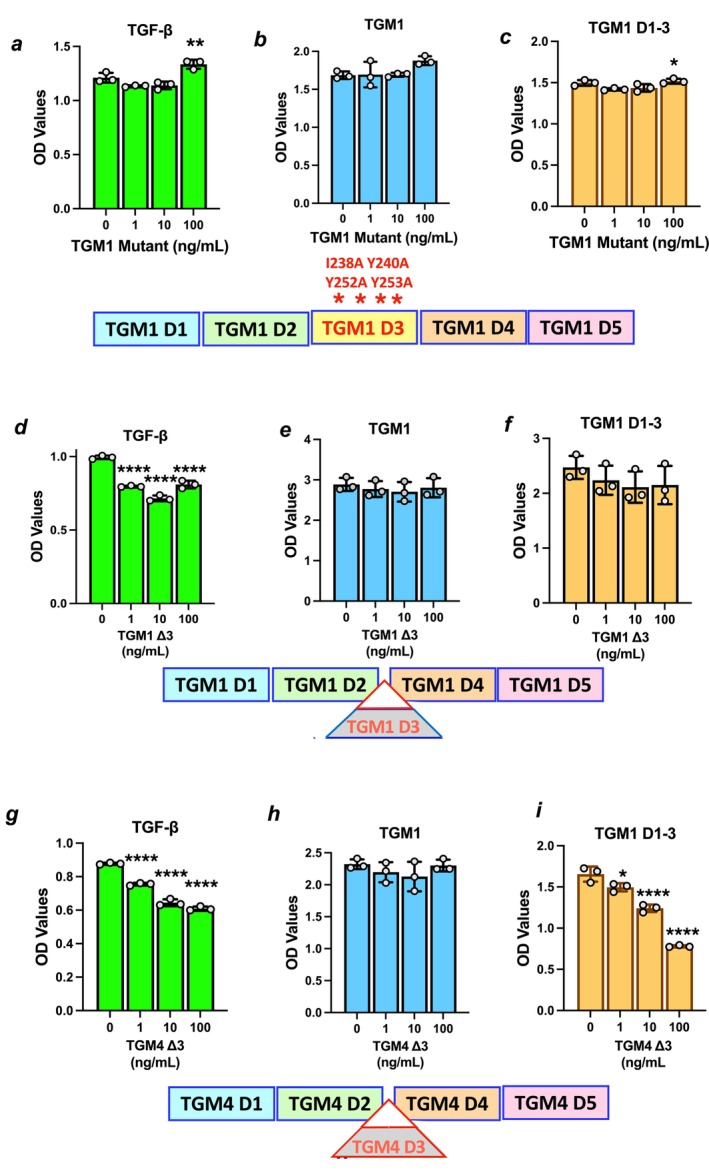
Deletion constructs as potential antagonists: ΔD1‐2 and ΔD3. (a–c) Test of mutant TGM1 with 4 amino acid alterations in D3 (I238A, Y240A, Y252A, and Y253A) to inhibit signaling of MFB‐F11 transcriptional reporter fibroblasts by (a) 5 ng/mL TGF‐β; (b) 10 ng/mL full‐length TGM1; and (c) 50 ng/mL TGM1 D1‐3. (d–f) Ability of TGM1 Δ3 to inhibit signaling of MFB‐F11 transcriptional reporter fibroblasts by (a) 5 ng/mL TGF‐β; (b) 10 ng/mL full‐length TGM1; and (c) 50 ng/mL TGM1 D1‐3. (g–i) Ability of TGM4 Δ3 to inhibit signaling of MFB‐F11 transcriptional fibroblasts by (a) 5 ng/mL TGF‐β; (b) 10 ng/mL full‐length TGM1; and (c) 50 ng/mL TGM1 D1‐3. Data shown are from the median of 3 concentrations of potential inhibitors tested, and analyzed by ordinary one‐way ANOVA for significant differences from the no‐inhibitor control. *p < 0.05, **p < 0.01, ****p < 0.0001; non‐significant differences (*p* > 0.05) are not shown.

TGM4 shows ~100‐fold lower affinity than TGM1 for TGFBR2 through domain D3 (Figure S1b). We therefore asked whether altering TGM1 in D3 to ablate its binding to TGFBR2 would convert it into a TGM4‐like antagonist, using a mutant form of TGM1 in which TGFBR2 interactions have been abolished by a set of four aa substitutions [10]. This construct was tested for inhibition of MFB‐F11 signaling by TGF‐β (Figure 2a), wild‐type TGM1 (Figure 2b), and also a truncated protein, TGM1 D1‐3 that lacks the CD44 co‐receptor binding domains D4‐5, which previously were found to be weak agonists [13] (Figure 2c). However, the TGM1 construct with mutated D3 showed no antagonistic properties, even against the latter, weakest agonist.

In a further step, we removed D3 entirely, and we found that TGM1‐Δ3 showed a modest but significant ability to impede TGF‐β activation of MFB‐F11 cells (Figure 2d), but no inhibitory activity against full‐length TGM1 or its truncated form D1‐3 (Figure 2e,f). We also made an equivalent D3 deletion of TGM4. TGM4 lacking D3 (TGM4‐Δ3) showed significant antagonism against TGF‐β (Figure 2g), and although unable to block TGM1 (Figure 2h), it strongly inhibited TGM1 D1‐3 (Figure 2i). Notably, the greater antagonistic activity in the absence of D3 demonstrates that interaction with TGFBR2 is not essential, and that inhibition does not operate through blockade of that receptor. Mechanistically, it is possible that Δ3 constructs can sequester TGFBR1 and inhibit the association with TGFBR2 that is required for signal activation.

### 3.3 Testing Antagonism by Truncated Proteins

**FIGURE 3:**
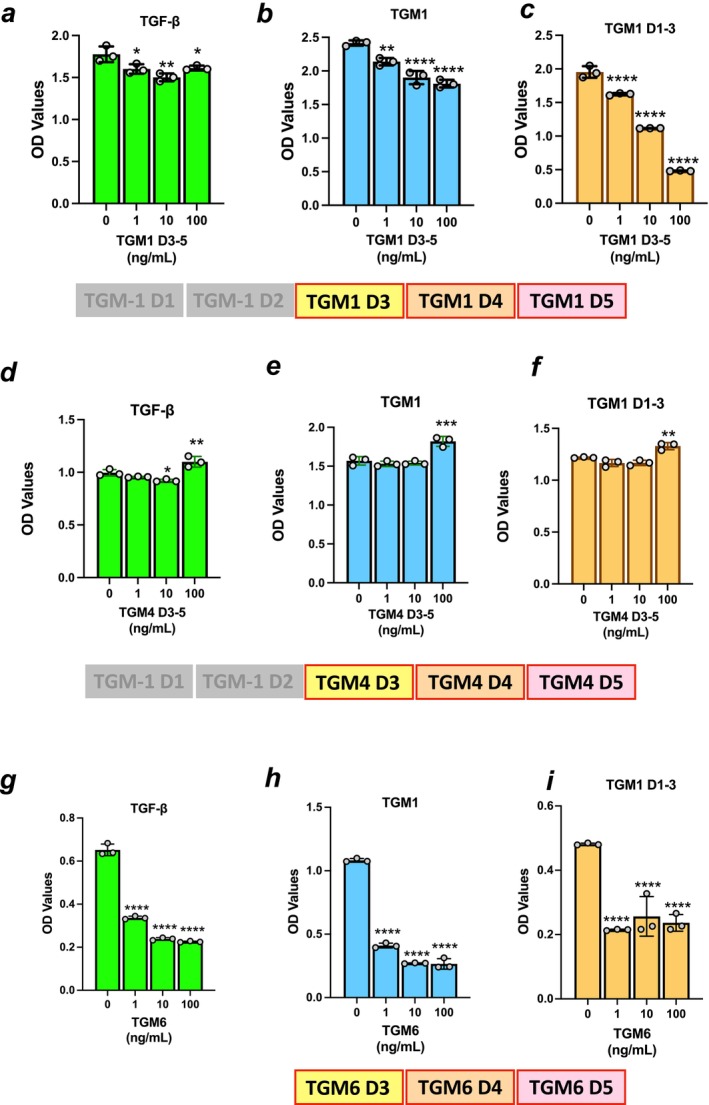
Analysis of truncated TGMs lacking D1‐2. (a–c) Ability of TGM1 D3‐5 to inhibit signaling of MFB‐F11 transcriptional reporter fibroblasts by (a) 5 ng/mL TGF‐β; (b) 10 ng/mL full‐length TGM1; and (c) 50 ng/mL TGM1 D1‐3. (d–f) As above, with TGM4 D3‐5 tested for ability to inhibit stimulation by (d) 5 ng/mL TGF‐β; (e) 10 ng/mL full‐length TGM1; and (f) 50 ng/mL TGM1 D1‐3. (g–i) As above, with TGM6 tested for ability to inhibit stimulation by (g) 5 ng/mL TGF‐β; (h) 10 ng/mL full‐length TGM1; and (i) 50 ng/mL TGM1 D1‐3. Data shown are from the median of three concentrations of potential inhibitors tested, and analyzed by ordinary one‐way ANOVA for significant differences from the no‐inhibitor control. *p < 0.05, **p < 0.01, ***p < 0.001, ****p < 0.0001; non‐significant differences (*p* > 0.05) are not shown.

We recently described a different member of the TGM family, TGM6, which lacks D12 domains and acts as an effective antagonist of TGF‐β signaling; in this protein, D3 binds TGFBR2 with higher affinity than is found for TGM1 or TGM4 [12]. To explore whether loss of D12 of TGM1 and TGM4 would replicate this inhibitory effect, we expressed Δ12 constructs of TGM1 and TGM4 containing only D3‐5, and tested them for antagonism of signaling induced by TGF‐β or TGM1 ligands. In the case of TGM1 D3‐5, only marginal degrees of reduction in signaling by TGF‐β were observed (Figure 3a), and modest decreases in TGM1 signaling (Figure 3b), although a more profound inhibition was seen with TGM1 D1‐3 (Figure 3c). Parallel studies with TGM4 D3‐5 failed to show any inhibition even of TGM1 D1‐3 (Figure 3d–f), which may be attributed to the much lower affinity of TGM4 D3 for TGFBR2 when compared with TGM1 D3. In contrast, and in accordance with earlier studies [12], TGM6 was able to profoundly suppress responses by each of the ligands tested (Figure 3g–i).

In the case of TGM4, we were also interested in determining if other domains were required for antagonism, using a set of truncation constructs previously described [15], and if either could act independently of D345. As shown in Figure S2, constructs lacking D5 were devoid of antagonism, demonstrating a requirement for the co‐receptor binding domain(s) for effective inhibition in fibroblasts.

### 3.4 Dimerization of TGM4 Confers Stimulatory Activity for Fibroblasts

**FIGURE 4:**
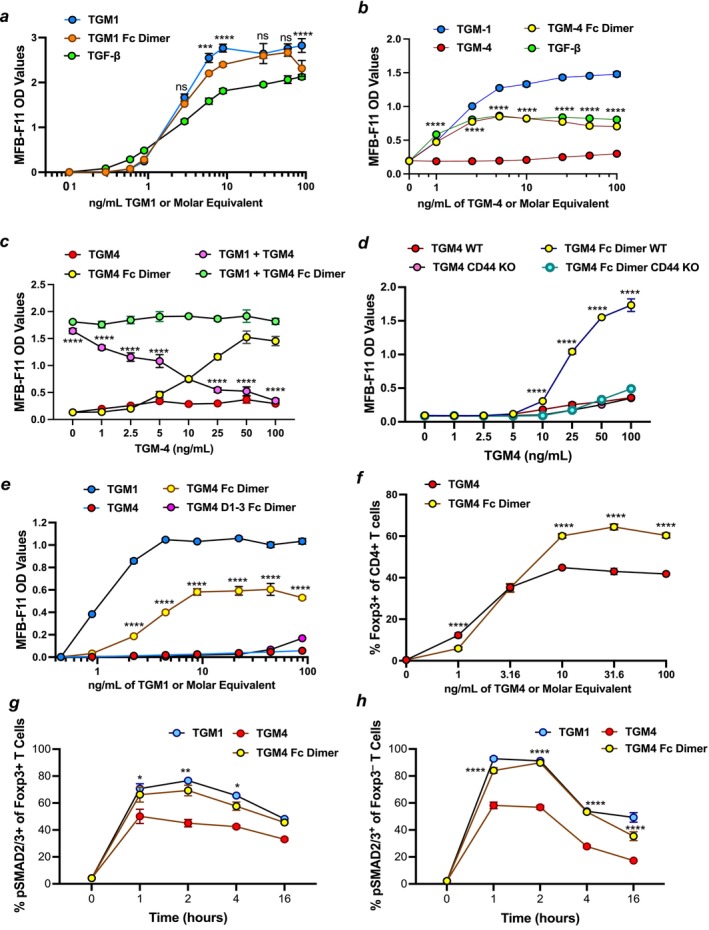
Dimerization of TGM4 enhances activation of fibroblasts and T cells. Molar equivalents used were based on monomeric ligand mol.wt of 49.1 kDa and Fc‐dimerized ligand mol.wt. of 144.7 kDa (Table S2), giving a ratio of 49.1:72.4, or 1.47 ng of Fc dimer per 1 ng of monomer. (a) TGM1‐Fc dimerization does not enhance its ability to activate SMAD3 transcriptional response in MFB‐F11 fibroblasts. Data are mean ± SD, *n* = 3 from one of two replicate experiments, analyzed by two‐way ANOVA with Sidák's multiple comparison test, showing statistics comparing TGM1 monomer and Fc dimer. ***p < 0.001; ****p < 0.0001; ns, not significant (*p* > 0.05). (b) TGM4‐Fc dimer activates SMAD3 transcriptional response in MFB‐F11 fibroblasts. Data are mean ± SD, *n* = 3 from one of three replicate experiments, analyzed by two‐way ANOVA with Sidák's multiple comparison test, showing statistics comparing TGM4 monomer and dimer. ****p < 0.0001. (c) SMAD3 transcriptional response assay in MFB‐F11 fibroblasts comparing monomeric TGM4 and TGM4‐Fc‐dimer, either alone or in addition to TGM1. Data are mean ± SD, *n* = 3 s, analyzed by two‐way ANOVA with Sidák's multiple comparison test, showing statistics comparing TGM4 monomer and dimer. ****p < 0.0001. (d) TGM4‐Fc dimer activation of SMAD3 transcriptional response is dependent on CD44 expression in MFB‐F11 cells. CD44 KO cells were constructed, as described previously [15]. Data are mean ± SD, *n* = 3 from one of two replicate experiments, analyzed by two‐way ANOVA with Sidák's multiple comparison test, showing statistics comparing TGM4 dimer responses in WT and CD44KO cells. ****p < 0.0001. (e) TGM4 D1‐3 dimer is unable to activate SMAD3 transcriptional response in MFB‐F11 fibroblasts in the same manner as full‐length TGM4 dimer. Data are mean ± SD, *n* = 3 from one of two replicate experiments, analyzed by two‐way ANOVA with Sidák's multiple comparison test, showing statistics comparing TGM4 full‐length and D1‐3 dimers. ****p < 0.0001. (f) TGM4 induction of Foxp3 expression in murine spleen CD4^+^ T cells by TGM4 in monomeric and dimeric forms. Data are mean ± SD, *n* = 3 from one of two replicate experiments, analyzed by two‐way ANOVA with Sidák's multiple comparison test. ****p < 0.0001. (g and h) SMAD2/3 phosphorylation of murine Foxp3^+^ (g) and Foxp3^−^ (h) T cells as analyzed by flow cytometry 1–16 h post‐stimulation with 100 ng/mL TGM1 or TGM4, or 139 ng/mL of TGM4‐Fc‐dimer (representing molar equivalent for binding sites). Data are mean ± SD, *n* = 2 from one of two replicate experiments, analyzed by two‐way ANOVA with Sidák's multiple comparison test. *p < 0.05; **p < 0.01;****p < 0.0001.

A conspicuous difference between members of the TGF‐β family and TGM proteins is that the former are dimerized through an interchain disulfide bond, whereas the latter are presumed to be monomeric with only intra‐domain disulfide bonds. To investigate whether multimerization affects TGM function, we made dimeric versions of the TGM proteins by fusion to human Fc (immunoglobulin fragment crystallizable) proteins. In the first instance, the dimeric Fc‐TGM1 construct was compared to the native TGM1 protein. However, in comparison, the dimerized TGM1‐Fc was slightly less potent (Figure 4a), suggesting that signaling by TGM proteins may not require assembly of the canonical heterotetrameric surface complex (two TGFBR1 and two TGFBR2 receptors). As the fusion partner may cause steric hindrance of binding sites, we also tested fusion proteins containing extended 15‐aa linkers, but found no difference (Figure S3a).

Next, we tested dimeric Fc fusion constructs of truncated versions of TGM1. As the absence of the CD44‐binding D4‐5 results in a 10‐fold loss of potency, we asked whether this could be restored by dimerization of TGM1 D1‐3. Surprisingly, the converse was observed, with a further loss of efficacy when fused to Fc (Figure S3b), which may be explained by the proximity of the fusion partner to the TGFBR2 binding residues near the C‐terminal of D3 [10]. Dimeric forms of D1‐2 or D3 (to cross‐link TGFBR1 or TGFBR2, respectively) lacked agonist activity as expected (Figure S3c), but also exhibited no ability to inhibit signaling by TGF‐β (Figure S3d,e).

We then prepared a dimeric TGM4‐Fc and tested it on MFB‐F11 fibroblasts. In marked contrast to the monomeric form, Fc‐dimerized TGM4 activated these SMAD3 transcriptional reporter cells (Figure 4b). Thus, Fc dimerization switched the functional phenotype of TGM4 from antagonist to agonist (Figure 4c). Signaling was fully dependent on the activity of the TGFBRs as the SMAD3 transcriptional response was inhibited by the addition of ITD1, which induces TGFBR2 degradation, SB431542, and SB525334 that inhibit TGFBR1 kinase activity, and SIS3 that interferes with SMAD3 function (Figure S4a). However, even when dimerized, TGM4 did not stimulate fibroblasts as strongly as TGM1, attaining a lower plateau level at maximal concentrations to the same degree as TGF‐β (Figure 4b). We also tested an alternative construct with a more extended linker between TGM4 and the Fc fusion partner, inserting an additional 15 aa at the junction site; this construct showed only a marginal increment in activity (Figure S4b).

To evaluate the role of co‐receptor interactions, the assays were repeated with MFB‐F11 cells that had been genetically modified to deplete CD44 expression [15]. In the absence of this co‐receptor, TGM4‐Fc dimer lost its ability to activate fibroblasts (Figure 4d), indicating that dimerization may enhance co‐receptor avidity rather than replicate the dimeric interactions of TGF‐β with the canonical receptors. Consistent with this finding, a Fc‐dimeric form of TGM4 limited to only D1‐3 was, like full‐length monomeric TGM4, unable to drive activation (Figure 4e).

We previously reported that although TGM4 is unable to stimulate fibroblasts, it can induce Foxp3 in murine T cells, although to a lesser degree than TGM1 [15]. Therefore, we compared monomeric and Fc‐dimeric TGM4 in T cells, measuring their induction of the Foxp3 transcription factor and the phosphorylation of SMAD2/3 by the TGFBR1 kinase. Foxp3 induction was elevated in cells treated with Fc‐dimeric TGM4, relative to those given monomeric form (Figure 4f). Flow cytometric analysis of phospho‐SMAD2/3 levels over 16 h post‐stimulation in both splenic Foxp3^+^ regulatory T cells and effector Foxp3^−^ T cells showed that in both subsets, monomeric TGM4 was markedly less potent than TGM1, but Fc‐dimerization enhanced the activity of TGM4 to a level comparable with monomeric TGM1 (Figure 4g,h).

### 3.5 Domain Swapping Experiments Demonstrate TGM4 D1‐2 Is Crucial for Inhibition

To investigate the roles of the different individual domains of TGMs in controlling activation and inhibition, we designed a set of domain‐swap constructs for expression in mammalian HEK293T cells, and tested purified TGM chimeric proteins in fibroblast, macrophage, and T cell‐based assays.

**FIGURE 5:**
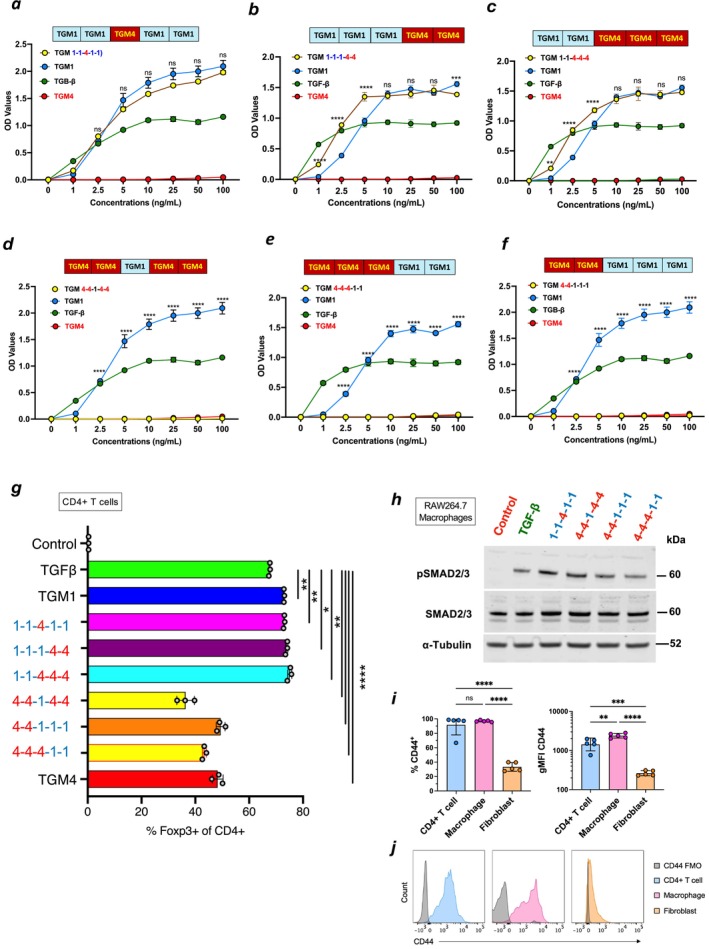
Activation of fibroblast signaling by domain swap constructs. (a–c) Analysis of TGM4 D3 (a), D45 (b), and D3‐5 (c) domain exchanges, assayed for activation of SMAD3 transcriptional response in MFB‐F11 fibroblasts, as measured by release of alkaline phosphatase. In each case, the chimeric protein is shown in yellow. Data are mean ± SD, *n* = 3 from one of two replicate experiments, with comparisons of TGM1 with the indicated chimera analyzed by two‐way ANOVA with Sidák's multiple comparison test. **p < 0.01;****p < 0.0001; ns, not significant (*p* > 0.05). (d–f) Analysis of TGM1 D3 (d), D45 (e), and D3‐5 (f) domain exchanges, assayed for activation of MFB‐F11fibroblasts. In each case, the chimeric protein is shown in yellow. Data are mean ± SD, *n* = 3 from one of two replicate experiments, with comparisons of TGM1 with the indicated chimera analyzed by two‐way ANOVA with Sidák's multiple comparison test. ****p < 0.0001. (g) Analysis of Foxp3 expression in mouse splenic T cells, cultured in anti‐CD3‐coated wells and incubated with 400 U/mL of IL‐2 and 100 ng/mL of the indicated ligands. Data are mean ± SD, *n* = 3 from one of two replicate experiments, analyzed by one‐way ANOVA with Dunnett's multiple comparisons test. *p < 0.05; **p < 0.01; ****p < 0.0001. (h) Western blot analysis of phospho‐SMAD (pSMAD) levels in lysates of RAW246.7 macrophages that were challenged by the indicated chimeric proteins, following incubation with 100 ng/mL of ligand for 1 h. α‐Tubulin, loading control. (i and j) Flow cytometric analyses of anti‐CD44 binding to small intestinal CD4^+^ T cells were (CD45^+^, CD3^+^CD4^+^CD19^−^), macrophages (CD45^+^CD11b^+^CD64^+^Ly6C^+^MHC‐II^+^), and fibroblasts (CD45^−^CD31^−^ESAM^−^Podoplanin^+^CD4^+^), presented as percentage positive and MFI (i) and as histograms (j). MFI, Mean Fluorescence Intensity; FMO, Fluorescence Minus One, that is, in the absence of anti‐CD44 antibody. Data presented are from five individual C57BL/6 mice analyzed by one‐way ANOVA; **p < 0.01; ****p < 0.0001.

In the first set of chimeras, we replaced D3 and/or D4‐5 of TGM1 with the corresponding modules from TGM4. The affinity of TGM4 D3 for TGFBR2 is around 100‐fold lower than that of TGM1 D3 [15]; when D3 of TGM4 was substituted into TGM1 (1‐1‐4‐1‐1), there was a slight diminution of activity that did not reach statistical significance (Figure 5a). This is consistent with previous work that had shown that mutations of TGM1 D3 that reduced affinity for TGFBR2 by more than 80‐fold only diminished potency to a small extent [15], with the co‐receptor domains evidently sufficing to provide sufficient avidity to enable binding and signaling. Conversely, substitution of the co‐receptor domains D4‐5 from TGM4 into TGM1 resulted in a modest (2‐fold) increase in potency that was highly significant when comparisons were made at low ligand concentrations of 1–5 ng/mL (Figure 5b). Similar enhancement of potency was observed when the D3‐5 tract of TGM1 was replaced by that of TGM4 (Figure 5c). Hence, the co‐receptor‐binding domains of TGM4 do not compromise the ability of a protein with TGM1 D1‐2 to activate signaling in fibroblasts.

We then tested the complementary set of chimeras. We found that none of the 3 constructs containing D1‐2 of TGM4 were able to activate MFB‐F11 fibroblasts, irrespective of whether they contained D3, D4‐5, or D3‐5 of TGM1 (Figure 5d–f, respectively), confirming that D1‐2 of the two TGM proteins determines the outcome of fibroblast stimulation. However, this result was counterintuitive as TGM4 has a 20‐fold higher affinity for TGFBR1 than the activating ligand TGM1 [15].

TGM4 has previously been reported to selectively induce TGF‐β signaling in immune cells such as macrophages and T cells [15]. To ascertain whether the chimeric proteins retained this activity, each was tested for the induction of the regulatory T cell‐specific transcription factor Foxp3 in primary murine splenocytes. As shown in Figure 5g, all constructs were able to induce expression, with those containing D12 of TGM1 driving higher levels than those with D12 of TGM4; earlier work had found that full‐length TGM4 is less potent than TGM1 in this assay [13], suggesting that again the differential properties between the two ligands are determined by D12.

We also tested the ability of all constructs to drive TGF‐β signaling in macrophages. Analyzing levels of phospho‐SMAD2/3 proteins in RAW264.7 cells demonstrated equally strong activation by all proteins irrespective of D12 composition (Figure 5h) and even at concentrations as low as 1.25 ng/mL (Figure S5a–d). This finding is in keeping with earlier work that TGM1 and TGM4 have equivalent potency on macrophage cell types [15].

As both TGM1 and TGM4 bind to CD44, we also compared expression levels of this co‐receptor on fibroblasts, macrophages, and T cells; small intestinal lamina propria cell suspensions were stained with cell type‐specific antibodies and anti‐CD44 and evaluated by flow cytometry; we found that fibroblasts express markedly lower levels of CD44 (Figure 5i,j), suggesting that interactions with TGM4 are below a key threshold for activation of these cells.

### 3.6 TGM4 D1‐2 Constructs Antagonize TGF‐β Signaling in Fibroblasts

**FIGURE 6:**
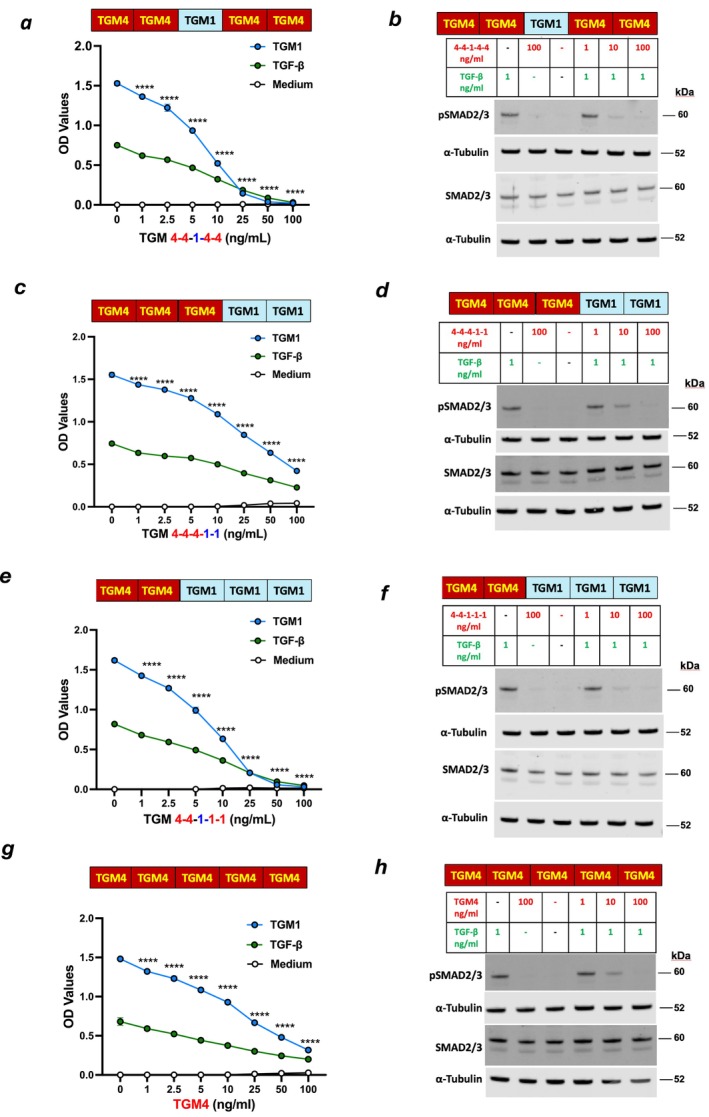
Domain swaps as potential antagonists. (a and b) Analysis of the 4‐4‐1‐4‐4 chimera D3 domain exchange for inhibitory effects on the SMAD activation response to 5 ng/mL TGF‐β or 10 ng/mL TGM1 in MFB‐F11 cells as measured by (a) release of alkaline phosphatase and (b) Western blot evaluation of SMAD2/3 phosphorylation levels in MFB‐F11 cell lysates.α‐Tubulin, loading control. (c and d) As a, b with the 4‐4‐4‐1‐1 chimera. (e and f) As a, b with the 4‐4‐1‐1‐1 chimera. (g and h) As a, b with native TGM4 control. Data represent mean ± SD (*n* = 3) for one of two replicate experiments, analyzed for differences between TGM1 response in the absence or presence of indicated concentrations of chimeras (a, c, and e) or native TGM4 (g) by two‐way ANOVA with Dunnett's multiple comparisons test.

As full‐length TGM4 can inhibit fibroblast activation by TGM1 and TGF‐β (Figure 1), we then tested all constructs containing D1‐2 of TGM4 to evaluate their capacity to interfere with signaling by TGF‐β and TGM1. Each construct with TGM4 D1‐2 together with TGM1 D3 (Figure 6a,b), D4‐5 (Figure 6c,d) or D3‐5 (Figure 6e,f) replacing the corresponding domains of TGM4 was as antagonistic as full‐length TGM4 in the same experiments (Figure 6g,h), measured both by reporter cell SMAD3/4 transcriptional activation (Figure 6a,c,e,g) and by SMAD2/3 phosphorylation (Figure 6b,d,f,h). The replacement of TGM4 D3 with that from TGM1 resulted in a more profound inhibition, irrespective of the D4‐5 status (co‐plotted in Figure S5e,f), indicating that, as TGM1 D3 has a higher affinity for TGFBR2 than TGM4 D3 does, the inability of TGM4 to activate fibroblasts cannot be explained simply by inadequate affinity for the TGFBR receptors. Hence, the effect of TGM4 is highly context‐dependent, with TGM4 D4‐5 enhancing activation when combined with D1‐2 of TGM1 (Figure 5b,c) and enhancing antagonism when combined with the same domains of TGM4 itself (Figure S5e,f).

## 4 Discussion

The family of TGF‐β mimic proteins from *H. polygyrus* presents a suite of novel agonists, such as TGM1, that fully replicates the effects of mammalian TGF‐β across several cell types. The gene family also includes antagonists (e.g., TGM6), which selectively block TGF‐β signaling in a co‐receptor‐dependent manner [11, 12, 13]. We earlier reported that TGM4 preferentially stimulates myeloid cells in a manner reliant on the co‐receptor CD44, but fails to signal in fibroblasts, thus representing a cell‐specific cytokine‐like ligand [15]. We now report that, unexpectedly, TGM4 acts in a bipolar fashion, antagonizing TGF‐β signaling in fibroblasts while activating immune cells through the same pathway. As TGF‐β is a major driver of fibrosis in many clinical settings [29, 30, 31], the discovery of a novel fibroblast‐specific inhibitor of TGF‐β signaling offers an exciting new lead in the search for therapies aimed at blocking fibrosis in vivo [32].

TGM1 and TGM4 each comprise 5 modular domains, with D1‐2 binding TGFBR1 and D3 binding TGFBR2, albeit with markedly different affinities (Figure S1a), whereas D4‐5 binds a co‐receptor, CD44 [10, 15, 16]. In recent studies, we reported that another family member, TGM6, which lacks D1‐2 and so is unable to recruit TGFBR1, is a potent antagonist [12]. However, this mechanism cannot explain the antagonism by TGM4, which displays a very high‐affinity interaction between its D1‐2 and TGFBR1 [15]. Through domain mutation, deletion, dimerization, and recombination, we have dissected the structural requirements for agonism and antagonism and studied how differential activity is modulated by the contexts of cell type, ligand valency and avidity, and the role of the 3 identified receptors, TGFBR1, TGFBR2, and CD44.

By domain‐swapping experiments, we established that the TGFBR1‐binding domains D1‐2 of TGM4 are instrumental in antagonism. Furthermore, replacing these with D1‐2 of TGM1 switches the protein to an activation phenotype despite its *lower* affinity for TGFBR1, indicating that antagonism occurs by binding of TGM4 to TGFBR1, aided by interactions with CD44 through D4‐5, forming a complex that—in fibroblasts but not in immune cells—does not transduce a signal. We noted also that CD44 levels are much lower on fibroblasts than immune cell types and suggest that this may impact the formation of complexes that are either agonistic or antagonistic, with the latter favored by D1‐2 of TGM4. Resolution of this question awaits structural analysis of such complexes.

The absence of D3 binding to TGFBR2 greatly reduces antagonism, suggesting that TGFBR2 is recruited to this complex, which is counterintuitive as activation occurs typically when TGFBR1 and TGFBR2 are part of heteromeric complexes. A further unexpected finding is that dimerization of TGM4 converts it into an activator of fibroblast signaling, suggesting either that there is an avidity threshold that is overcome by dimerization or that the dimeric complex is qualitatively different from that formed by the native protein.

We have considered whether there is an inhibitory co‐receptor on fibroblasts that TGM4 D1‐2 engages, although this possibility appears to be excluded by the agonism of dimerized TGM4‐Fc. Antagonism also requires the continual presence of TGM4, arguing against a model involving a negative signal from another receptor. Furthermore, although previous pull‐down analysis showed that CD49b and CD206 are significant interactors with TGM4 [15], these co‐receptors were found on myeloid cells (which TGM4 activates) but not fibroblasts (which it inhibits) and hence are unlikely intermediaries in this process. However, it may be that an inhibitory complex is formed only in the absence of one of these receptors, such as CD49d, which was shown to interact with D1‐3 of TGM4 [15]. It is also possible that there are additional TGM4 co‐receptors or other surface proteins that are known to interact with or inhibit TGFBR signaling [33, 34, 35] and these may be differentially expressed in different cell types, again influencing the outcome of TGM4 ligation.

In addition to the differential expression of co‐receptors by different cell types and the variable levels of CD44 found on immune and non‐immune cells, there may also be a significant disparity in the density of the two TGFBRs in different cell types that could relate to the impact of TGM4, and/or in the distribution of receptors in membrane domains such as lipid rafts. For example, it was recently reported that among > 1000 cell line transcriptomes, levels of TGFBR1 and TGFBR2 could vary independently by more than an order of magnitude [36]. A further factor is that the association of these receptors with cholesterol‐rich lipid rafts has been reported to limit and degrade TGF‐β signaling [37]. Taken together, these factors raise new questions about the environmental context of TGFBRs on different cell types, and whether understanding these contexts can facilitate cell‐specific manipulation of the signaling pathway.

As mentioned previously, *H. polygyrus* also elaborates a different TGM family member, TGM6, which inhibits TGF‐β signaling through interactions with TGFBR2 rather than TGRBR1 [12]. It seems remarkable that one helminth species should have evolved parallel mechanisms of manipulating this pathway in its host, raising the broader biological question of how this would advantage the parasite. In mice, its life cycle includes an initial breach of the intestinal barrier, followed by larval encystment in submucosal tissue of the small intestine, and, 1 week later, exit into the lumen through further epithelial breaches [38]. One possibility is that although dampening immune reactivity through TGF‐β signaling allows persistence in the host [39], inhibiting fibrosis may pre‐empt larval trapping in submucosal granulomas and/or protect the intestinal environment in which the adult parasites reside for long periods.

We are now seeking to resolve the TGM4 D1‐2:TGFBR1 and D4‐5:CD44 structures, to complement the known D3:TGFBR2 complex, and elucidate how agonism and antagonism by the closely related TGM ligands may operate. In particular, we seek to understand why the threshold for fibroblast stimulation by TGM4 is so much higher than it is for TGM1, and what implications this may have for receptor complex assembly and activation. We anticipate that these insights will allow us to engineer TGMs to regulate TGF‐β signaling across a range of settings, guided by and leveraging the evolutionary adaptations of a parasite with novel molecular structures for immune modulation.

## Author Contributions

Kyle T. Cunningham and Rick M. Maizels conceived and designed the research; Kyle T. Cunningham, Claire Ciancia, Tiffany Campion, Maarten van Dinther, Nadia Davis, Anja Duffy, Anna L. L. Heawood, Luke Power, Anna Sanders, Shashi P. Singh, Elizabeth Thompson and Ruby White performed the research and acquired the data. Kyle T. Cunningham, Andrew P. Hinck, Peter ten Dijke and Rick M. Maizels analyzed and interpreted the data. All authors were involved in drafting and revising the manuscript.

## Funding

This work was supported by Wellcome Trust (WT) (104111, 306173, and 228330) and National Institutes of Health (Grant R03 AI153915).

## Conflicts of Interest

The authors declare no conflicts of interest.

## Supporting information


**Figures S1–S5:** fsb271338‐sup‐0001‐Figures S1–S5.docx.

## Data Availability

The data supporting the findings reported in this paper are openly available from the Senior Authors and from the repositories detailed in the Methods section.
