# Supplementary material for: Molecular Engineering of the Helminth TGF‐β Mimetics, TGM1 and TGM4, Reveals a Novel Antagonist of TGF‐β Signaling in Fibroblasts
Source: FASEB J. 2026 Mar 31;40(7):e71338. doi: 10.1096/fj.202503194R (PMC13037750; doi:10.1096/fj.202503194R)
Supplement: Supplementary file 1 — Figures S1–S5: fsb271338‐sup‐0001‐Figures S1–S5.docx. [file FSB2-40-e71338-s001.docx]

**Cunningham Supplementary Material 2025 12 15**

**Molecular engineering of the helminth TGF-β mimetics, TGM1 and TGM4 reveals a novel antagonist of TGF-β signaling in fibroblasts**

Kyle T. Cunningham^1,†^, Claire Ciancia^1^, Tiffany Campion^1,‡^, Maarten van Dinther^2^, Nadia Davis^1^, Anja Duffy^1^, Anna L. L. Heawood^1^,Luke Power^1^, Anna Sanders^1^, Shashi P. Singh^1¶^, Danielle J. Smyth^1,∑^, Elizabeth Thompson^1^, Ruby White^1^, Andrew P. Hinck^3^, Peter ten Dijke^2^ and Rick M. Maizels^1^*

**Supplementary Figures**

**Figure S1. Sequence and affinity comparisons of TGM1 and TGM4.**

**a.** Amino acid (aa) alignments of D1 and D2. Identical residues are in shaded boxes.

**b.** Schematic of the 5 domains of TGM1 and TGM4, with amino acid identities in % for individual domains shown in each colored box. Binding affinities of D1-2 for TGFBR1, D3 for TGFBR2 and D4-5 for CD44 are shown, as determined previously (10, 15).

**Figure S2 Responses to truncated TGM4 constructs**

SMAD3 transcriptional response in MFB-F11 fibroblasts to TGF-β (5 ng/mL), TGM1 (10 ng/mL) and TGM1 D1-3 (50 ng/mL) in the presence of increasing concentrations of TGM4 D1-2 (a), D1-3 (b) and D1-4 (c). Data shown are mean ± SD of 3 technical replicates.

**Figure S3 Responses to Fc dimeric constructs of TGM1 and its truncations**

**a.** SMAD3 transcriptional response in MFB-F11 fibroblasts to TGM1 dimer without or with extended linker (3x GGGGS) separating TGM1 from Fc.

**b.** SMAD3 transcriptional response in MFB-F11 fibroblasts to TGM1 D1-3 Fc dimer compared to D1-3 and full-length monomers.

**c.** Lack of SMAD3 transcriptional response MFB-F11 in fibroblasts to TGM1 D1-2 and D3 Fc dimers.

**d, e** TGM1 D1-2 and D3 Fc dimers do not inhibit SMAD3 transcriptional response in MFB-F11 fibroblasts to TGF-β.

Data shown are mean ± SD of 3 technical replicates.

**Figure S4. Responses to dimeric constructs of TGM4 and its truncations**

**a.** TGF-β signaling inhibitors (ITD1, a promoter of TGFBR2 degradation, SB4231542 and SB525334, two TGFBR1-like kinase inhibitors and SIS3, a SMAD3 small molecule inhibitor) ablate Fc-dimer TGM4 activity on SMAD3 transcriptional response in MFB-F11 fibroblasts.

**b.** SMAD3 transcriptional responses of MFB-F11 fibroblasts to TGM4 dimer without or with extended linker (3 x GGGGS) separating TGM4 from Fc and TGM4 monomer.

**c,d.** TGM4 D12 does not activate (**c**) or inhibit (**d**) SMAD3 transcriptional response in MFB-F11 fibroblasts.

**Figure S5. Comparisons of chimeric constructs in fibroblast responses**

**a-d** Induction of SMAD2 phosphorylation in RAW247 macrophages by the indicated TGM1/4 chimeras as measured by densitometry of Western blots of cell lysates, normalised to the response to 5 ng/mL TGF-β (set as 1.0).

**e, f** Re-plotting of data in Figure 3 a,c,e,g to show relative efficacy of the indicated doses of different chimeras in blocking SMAD3-induced transcriptional responses to 5 ng/mL TGF-β (**e**) and 10 ng/mL TGM1 (**f**).

|  | **aa** | **TGM1** | **TGM4** |
| --- | --- | --- | --- |
| **D1** | 19-95* | GCMPFSDEAATYKYVAKGPKNIEIPAQIDNSGMYPDYTHVKRFCKGLHGEDTTGWFVGICLASQWYYYEGVQECDDR | GCMPFSDETASYKYLTERSRNDETPAQNDSSGAYPDHTHVKRFCKGLHGEEKTGRYVGICLGSEWVYYQGVQECQDR |
| **D2** | 96-176 | RCSPLPTNDTVSFEYLKATVNPGIIFNITVHPDASGKYPELTYIKRICKNFPTDSNVQGHIIGMCYNAEWQFSSTPTCPAS | RCSPLPTNDTVTYEYLKATVNAGINFNITVHPDASGKYPELTYIKRICKNFPADSKVQGHIIGMCYNAEWRFSSTPTCPPS |
| **D3** | 177-262 | GCPPLPDDGIVFYEYYGYAGDRHTVGPVVTKDSSGNYPSPTHARRRCRALSQEADPGEFVAICYKSGTTGESHWEYYKNIGKCPDP | GCPPLPDDGIVFYEYYGYAGNRHTVGRAVSKDSSGNYPPQTHARRRCRALSQKADPGEFVGICYKSGTTGESHWDYYSHIRKCPDP |
| **D4** | 263-343 | RCKPLEANESVHYEYFTMTNETDKKKGPPAKVGKSGKYPEHTCVKKVCSKWPYTCSTGGPIFGECIGATWNFTALMECINA | RCKPLETNVSVHYEYFTMTNETGRKEGTPAEVDKGGKYPQHTCVRKFCDKSPYTCSVKGPIFGECLDGQWNFTALDECLNA |
| **D5** | 344-422 | RGCSSDDLFDKLGFEKVIVRKGEGSDSYKDDFARFYATGSKVIAECGGKTVRLECSNGEWHEPGTKTVHRCTKDGIRTL | RGCNSDDLFDKLGFEGVMVREEEGSDSYKDDFVRFYATGSKVNAECKGKTVQLECSDGEWHDPGTKTVHRCTKEGIRAL |

**Supplementary Table 1. Amino acid sequences of each of 5 domains of TGM1 and TGM4.** * Note that D1 constructs include additional N-terminal amino acids predicted to be in native proteins following signal peptide cleavage, for TGM1 aa16-18 (DDS), and for TGM4 aa 18 (S); variants in these were found to have identical functional properties.

|  | **Amino acids (aa)** | **Vector and restriction sites** | **Mol wt**  **Da** | **1 nM in ng/ml** | **1 µg/mL in nM** | **Clone Ref No.** |
| --- | --- | --- | --- | --- | --- | --- |
| **TGM1 Constructs** | | | | | | |
| TGM1 | aa 16-424 | pSecTag2A *Asc*I-*Apa*I | 48,485 | 48.5 | 20.6 | YH-039 |
| TGM1 Fc Dimer | aa 16-424+ HuIgG1Fc | pFuse *Eco*RI-*Nco*I | 142,843 | 71.4 * | 14.0 * | TC-007 |
| TGM1 FcDimer | aa 16-424+15 aa linker + HuIgG1Fc | pFuse *Eco*RI-*Nco*I | 144,248 | 72.1 * | 13.9 * | TC-037 |
| TGM1 D1-2 Fc Dimer | aa 16-176+ HuIgG1Fc | pFuse *Eco*RI-*Nco*I | 88,374 | 44.2 * | 22.6 * | TC-009 |
| TGM1 D1-3 Fc Dimer | aa 16-262+ HuIgG1Fc | pFuse *Eco*RI-*Nco*I | 107,345 | 53.7 * | 18.6 * | TC-008 |
| TGM1 D3 Fc Dimer | aa 177-262+ HuIgG1Fc | pFuse *Eco*RI-*Nco*I | 71,252 | 35.6 * | 28.1 * | TC-010 |
| TGM1 D1-3 | aa 16-262 | pSecTag2A *Asc*I-*Apa*I | 30,750 | 30.8 | 32.5 | ETB-002 |
| TGM1 D3-5 | aa 177-424 | pSecTag2A *Asc*I-*Apa*I | 30,439 | 30.4 | 32.9 | IS-003 |
| TGM1 Δ3 | aa 16-166 + 263-422 | pSecTag2A *Asc*I-*Not*I | 39,811 | 39.8 | 25.1 | TC-055 |
| TGM1 Mutant D3 | aa 16-424 (I238A,Y240A,Y252A,Y253A) | pSecTag2A *Asc*I-*Not*I | 48,978 | 49.0 | 20.4 | TC-054 |
| **TGM4 Constructs** | | | | | | |
| TGM4 | aa 18-424 | pSecTag2A *Asc*I-*Not*I | 49,060 | 49.1 | 20.4 | JN-005 |
| TGM4 Fc Dimer | aa 18-424+ HuIgG1Fc | pFuse *Eco*RI-*Nco*I | 143,309 | 71.7 * | 14.0 * | TC-011 |
| TGM4 Fc Dimer | aa 18-424+ 15 aa linker + HuIgG1Fc | pFuse *Eco*RI-*Nco*I | 144,712 | 72.4 * | 13.8 * | TC-038 |
| TGM4 D1-2 Fc Dimer | aa 18-176+ HuIgG1Fc | pFuse *Eco*RI-*Nco*I | 88,205 | 44.1 * | 22.7 * | TC-012 |
| TGM4 D1-3 Fc Dimer | aa 18-262+ HuIgG1Fc | pFuse *Eco*RI-*Nco*I | 107,034 | 53.5 * | 18.7 * | LP-008 |
| TGM4 D1 | aa 18-95 | pSecTag2A *Asc*I-*Apa*I | 12,485 | 12.5 | 80.1 | DJS-048 |
| TGM4 D1-2 | aa 18-176 | pSecTag2A *Asc*I-*Apa*I | 21,538 | 21.5 | 46.4 | DJS-047 |
| TGM4 D1-3 | aa 18-262 | pSecTag2A *Asc*I-*Not*I | 31,103 | 31.1 | 32.2 | DJS-046 |
| TGM4 D1-4 | aa 18-343 | pSecTag2A *Asc*I-*Not*I | 40,227 | 40.2 | 24.9 | DJS-045 |
| TGM4 D2-5 | aa 96-424 | pSecTag2A *Asc*I-*Apa*I | 39,794 | 39.8 | 25.1 | DJS-041 |
| TGM4 D3-5 | aa 177-424 | pSecTag2A *Asc*I-*Apa*I | 30,771 | 30.8 | 32.5 | DJS-042 |
| TGM4 D4-5 | aa 263-424 | pSecTag2A *Asc*I-*Not*I | 21,618 | 21.6 | 46.3 | AS-018 |
| TGM4 D5 | aa 344-422 | pSecTag2A *Asc*I-*Not*I | 12,464 | 12.5 | 80.2 | DJS-044 |
| TGM4 Δ3 | aa 18-166 + 263-422 | pSecTag2A *Asc*I-*Not*I | 39,935 | 39.9 | 25.0 | TC-056 |
| **TGM1/4 Chimeras** | | | | | | |
| 1-1-1-4-4 | aa 16-424 | pSecTag2A *Asc*I-*Not*I | 49,119 | 49.1 | 20.4 | CC-032 |
| 1-1-4-1-1 | aa 16-424 | pSecTag2A *Asc*I-*Apa*I | 48,595 | 48.6 | 20.6 | TC-058 |
| 1-1-4-4-4 | aa 16-424 | pSecTag2A *Asc*I-*Apa*I | 48,817 | 48.8 | 20.5 | CC-031 |
| 4-4-4-1-1 | aa 18-424 | pSecTag2A *Asc*I-*Not*I | 48,838 | 48.8 | 20.5 | AD-002 |
| 4-4-1-4-4 | aa 18-424 | pSecTag2A *Asc*I-*Not*I | 48,950 | 49.0 | 20.4 | TC-057 |
| 4-4-1-1-1 | aa 18-424 | pSecTag2A *Asc*I-*Not*I | 48,728 | 48.7 | 20.5 | TC-028 |

**Supplementary Table 2 : Proteins and Constructs used in this Study, with molecular weights and molarities.** * Molarity calculated per binding site (Fc dimer is bivalent). pSecTag expressed proteins include vector-encoded leader sequence (MGTDTLLLWVLLLWVPGSTG, cleaved from mature protein) and 5’ multiple cloning site (encoding DAANPPG), plus 3’ multiple cloning site, myc epitope and polyhistidine tag; pSecTag N terminal, *Not*I : AARGGPENKLISEEDLNSAVDHHHHHH (27 aa, 3,008 Da). pSecTag N terminal *Apa*I : PENKLISEEDLNSAVDHHHHHH (22 aa, 2,596 Da); pFUSE expressed proteins include vector-encoded IL-2 leader sequence (aa 1-23, ﻿MYRMQLLSCIALSLALVTNSISA, cleaved from mature protein), 4 aa from the multiple cloning site (MVRS, 483 Da), with or without 3xGGGGS linkers (15 aa, 964 Da), and human Fc (227 aa, 25,580 Da). All plasmids are available on request from the Maizels laboratory, by quoting the clone reference number.
